# Enantioselective Protonation of Radical Anion Intermediates in Photoallylation and Photoreduction Reactions of 3,3-Diaryl-1,1-dicyano-2-methylprop-1-ene with Allyltrimethylsilane

**DOI:** 10.3390/molecules24142677

**Published:** 2019-07-23

**Authors:** Hajime Maeda, Masayuki Iida, Daisuke Ogawa, Kazuhiko Mizuno

**Affiliations:** Department of Applied Chemisty, College of Engineering, Osaka Prefecture University, 1-1 Gakuen-cho, Naka-ku, Sakai, Osaka 599-8531, Japan

**Keywords:** photoreaction, photoinduced electron transfer, photoredox catalyst, Felkin-Anh model, radical anion, electron deficient alkene, allylsilane, mandelic acid, enantioselectivity, enantiomer

## Abstract

Photoreactions of acetonitrile solutions of 3,3-diaryl-1,1-dicyano-2-methylprop-1-enes (**1a**–**c**) with allyltrimethylsilane (**2**) in the presence of phenanthrene as a photoredox catalyst and acetic acid as a proton source formed photoallylation (**3**) and photoreduction (**4**) products via photoinduced electron transfer pathways. When (*S*)-mandelic acid was used as the proton source, the reactions proceeded with 3.4 and 4.8 %ee for formation of **3** and **4**, respectively. The results of studies of the effect of aryl ring substituents and several chiral carboxylic acids suggested that the enantioselectivities of the reactions are governed by steric controlled proton transfer in intermediate complexes formed by π-π and OH-π interactions of anion radicals derived from **1a**–**c** and chiral carboxylic acids.

## 1. Introduction

**Scheme 1:**
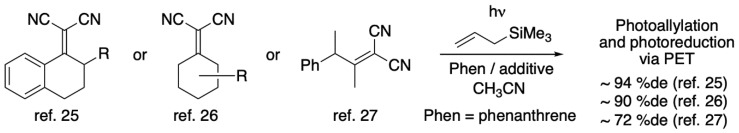
Our previous works.

Coupling reactions proceeding through photoinduced electron transfer (PET) pathways have been extensively studied from both a synthetic as well as a mechanistic viewpoint [1,2,3,4,5,6,7,8,9,10,11,12,13,14]. Because radical ions that serve as intermediates in these processes are short-lived and highly reactive, control of the stereochemistry of these reactions is often difficult [15,16,17,18,19,20,21]. We have previously developed photoallylation and photoreduction reactions of electron deficient alkenes with allyltrimethylsilane that occur via PET pathways [22,23,24]. In addition, we also demonstrated that diastereoselectivity of this process can be achieved by steric control of allyl radical or proton addition to radical anions that are generated from electron deficient alkenes (Scheme 1) [25,26,27]. The current study was aimed at the development of enantioselective PET promoted coupling reactions, and specifically, at assessing the effect of chiral carboxylic acids on the stereochemical outcomes of photoallylation and photoreduction reactions of prochiral electron deficient alkenes. The results showed that these processes took place with maximum 3.4–4.8 %ee when (*S*)-mandelic acid was used as the chiral proton source.

## 2. Results and Discussion

**Scheme 2:**
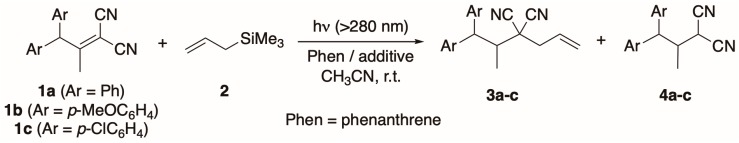
Photoallylation and photoreduction of **1a**–**c** by using allyltrimethylsilane (**2**).

Irradiation of an acetonitrile solution containing 1,1-dicyano-2-methyl-3,3-diphenylprop-1-ene (**1a**), 3 equiv of allyltrimethylsilane (**2**), a catalytic amount of phenanthrene (Phen) as a photoredox catalyst in a Pyrex vessel using a 300 W high-pressure mercury lamp was found to produce reduction product **4a** in 53% yield (Scheme 2, Table 1, entry 1, supplementary). Photoreaction of **1a** with **2** in the presence of acetic acid produced the allylated product **3a** in addition to **4a** in 34 and 31% yields, respectively (entry 2). The corresponding products **3b**–**c** and **4b**–**c** were produced in photoreactions of bis(*p*-methoxyphenyl) (**1b**) and bis(*p*-chlorophenyl) (**1c**) derivatives conducted under the same conditions (entries 3–6). The irradiation times used for these processes are those required for complete consumption of **1a**–**c**. The observed efficiencies of the reactions, based on the required irradiation times, decreased in the order **1c** > **1a** > **1b**.

**Figure 1:**
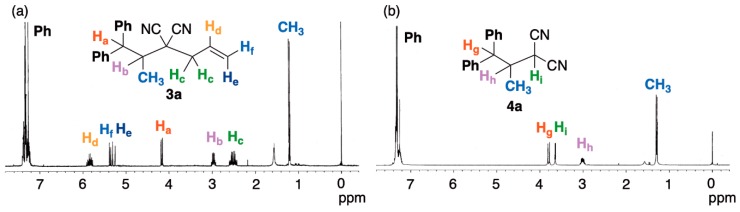
300 MHz ^1^H-NMR spectra of **3a** and **4a** in CDCl_3_.

**Scheme 3:**
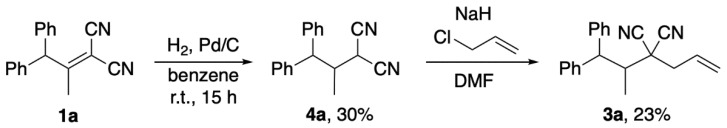
Synthesis of authentic samples of **3a** and **4a**.

Structures of photoproducts **3a**–**c** and **4a**–**c** were determined by using spectroscopic methods. In ^1^H-NMR spectra of CDCl_3_ solutions of **3a** and **4a** (Figure 1), the chemical shifts of resonances for protons that are bonded to the asymmetric carbons, i.e., H_b_ in **3a** and H_h_ in **4a**, were 2.97 (qd) and 3.02 (qt) ppm, respectively. Authentic samples of the photoproducts were prepared by hydrogenation of **1a** using Pd/C to form **4a** and ensuing allylation of **4a** using allyl chloride to form **3a** (Scheme 3). The spectral data for the synthesized compounds were identical to those of photoproduced **3a** and **4a**.

**Figure 2:**
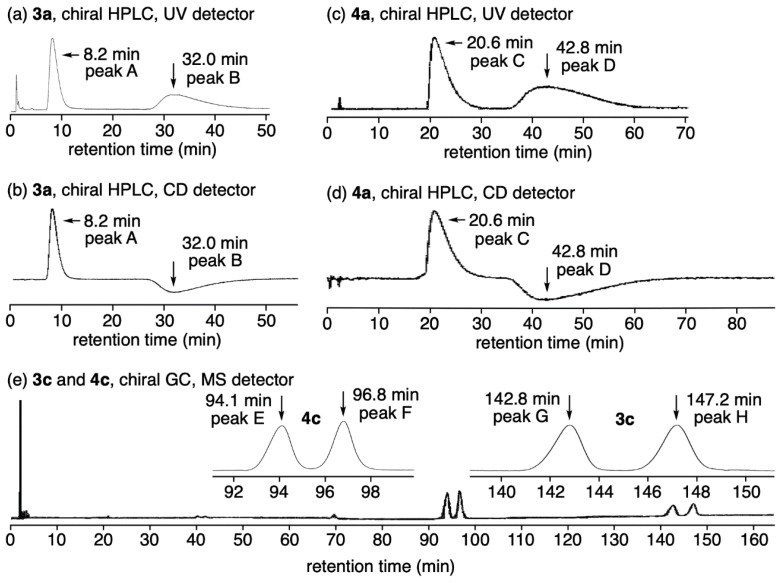
Resolution of the enantiomers of (**a**,**b**) **3a** by using chiral HPLC with UV and CD detectors both at 270 nm, (**c**,**d**) **4a** by chiral HPLC with UV and CD detectors both at 270 nm, and (**e**) **3c** and **4c** by chiral GC with a MS detector.

In order to explore the enantioselectivities of these photoreactions, samples of **3a** and **4a** were subjected to HPLC using a chiral stationary phase with the effluents being monitored by using UV and CD detectors (Figure 2a–d). The results showed that two peaks in the HPLC trace for the enantiomers of **3a** and **4a** were completely resolved. Unfortunately, HPLC conditions could not be found for resolution of the enantiomers of **3b** and **4b**. Moreover, the enantiomers of **3c** and **4c** can be separated by using GC with a chiral capillary column (Figure 2e).

**Figure 3:**
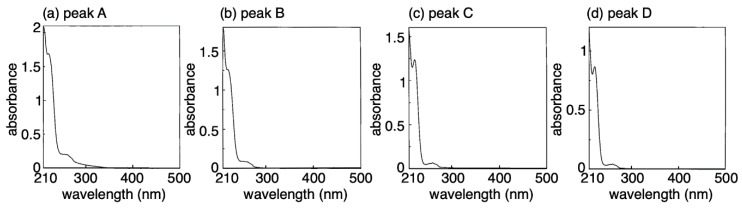
UV-vis absorption spectra of ethanol solutions of substances from (**a**) peak A, (**b**) peak B, (**c**) peak C, and (**d**) peak D.

**Figure 4:**
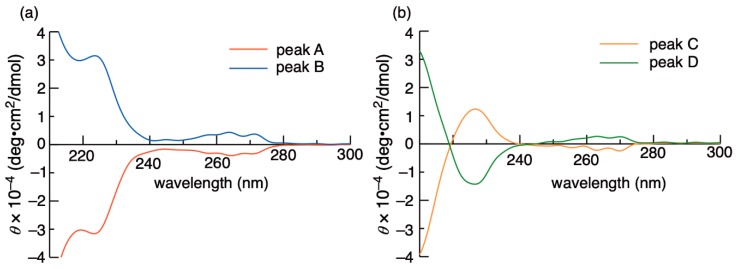
Circular dichromism (CD) spectra of ethanol solutions of substances from (**a**) peaks A and B, (**b**) peaks C and D.

In order to prove that these separation techniques led to the individual enantiomers, the effluents of peaks A–D in Figure 2a–d were collected, concentrated in vacuo and the residues in ethanol were subjected to UV-vis absorption and CD spectroscopic analysis (Figure 3 and Figure 4). The UV-vis absorption spectra of substances in effluents corresponding to peaks A and C were identical to those from peaks B and D, respectively. In addition, ^1^H-NMR and mass spectra of the respective substances in peaks A and C were also identical to those in peaks B and D, respectively. Moreover, CD spectral traces of substances comprising peaks A and B, and peaks C and D, respectively, were mirror images relative to the horizontal base line. The combined results indicated that the enantiomers of these substances can be resolved by using chromatographic methods.

To assess the potential of introducing enantioselectivity into the photoreactions described above, irradiations were carried out on solutions of **1a**–**c** and allyltrimethylsilane (**2**) containing chiral carboxylic acids. The yields and percent enantiomeric excesses (%ee) of products formed in these processes are listed in Table 2. The %ee in each case was calculated using the ratio of areas under the chiral HPLC or GC peaks corresponding to the enantiomers as %ee when acetic acid was used becoming zero. A positive %ee value corresponds to a situation in which the major isomer is the second peak, while a negative value shows that the major isomer is the first peak. The absolute structures could not be decided. The data arising from photoreactions in the absence or presence of achiral acetic acid are also included in Table 2 for comparison purposes.

Use of 1 equiv of (*R*)-mandelic acid in photoreaction of **1a** with **2** led to formation of **3a** and **4a** with respective +1.5 and +4.1 %ee values (entry 3). A reversal in major enantiomers of the products arose from the reaction of **1a** with **2** conducted in the presence of (*S*)-mandelic acid (entry 4), which supports the reaction proceeding in an enantioselective manner. Also, when l-lactic acid was used in this photoreaction, the major enantiomers were the reverse of those formed in reactions in the presence of (*S*)-mandelic acid (entry 5). The use of *C*_2_ symmetric dibenzoyl l-tartaric acid did not promote an increase of %ee of either product (entry 6). The photoreaction of **1b** with **2** also occurred when (*R*)- and (*S*)-mandelic acids were used, however the %ee of either product could not be determined (entries 9 and 10). Like in the case of **1a**, photoreaction of **1c** produced products **3c** and **4c** in which the major enantiomers were reversed when (*R*)- and (*S*)-mandelic acids were utilized (entries 13 and 14). Moreover, the results showed that the %ee improved up to 3.5 when (*S*)-2-(6-methoxy-2-naphthyl)propionic acid was used as a chiral acid (entry 15).

**Scheme 4:**
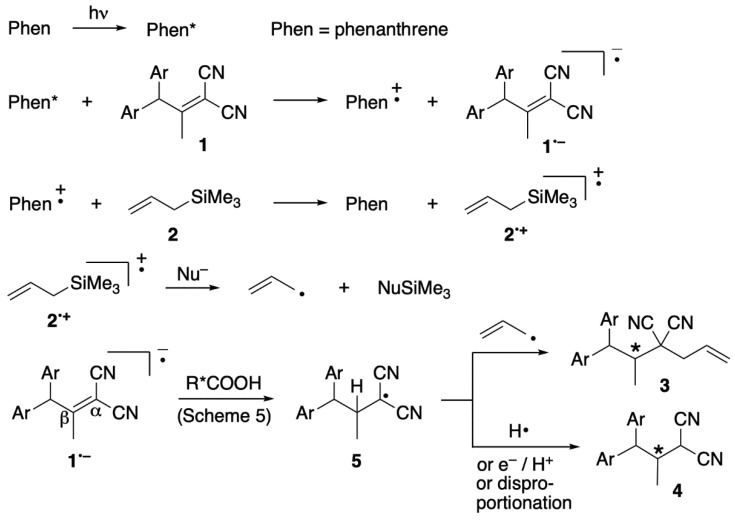
Mechanism for photoallylation and photoreduction of **1**.

Each photoreaction described above takes place through a process termed photoredox sensitization by phenanthrene (Phen) (Scheme 4) [22,23,24,25,26,27]. In the pathway, the excited singlet state of Phen, generated by light absorption, transfers one electron (SET (single electron transfer)) to the electron-deficient alkene **1** to form the phenanthrene radical cation (Phen^•+^) and the alkene radical anion **1**^•−^. The subsequent SET from allyltrimethylsilane (**2**) to Phen^•+^ generates recovered Phen and the radical cation **2**^•+^, which undergoes nucleophile-assisted Si-C bond cleavage [28,29,30] to form the allyl radical. Also, radical anion **1**^•−^ is protonated by the carboxylic acid to produce radical **5**, which upon coupling with the allyl radical generates the allylation product **3**. In a competitive pathway, radical **5** undergoes hydrogen abstraction or one-electron reduction followed by protonation or disproportionation to form reduction product **4** [23]. The inefficiency of the photoreaction of the MeO-substituted substrate **1b** and high efficiency of the reaction of Cl-substituted reactant **1c** are likely consequences of the stabilities of the corresponding radical anions **1b**^•–^ and **1c**^•–^ which governs their rates of formation by SET from relative to unproductive decay of the excited singlet state of Phen.

Based on the results of molecular orbital calculations with related compounds, it is estimated that the radical contribution to radical anion **1**^•–^ is large at the dicyano substituted carbon (α) and that negative charge density is large at the dialkyl substituted carbon (β) [23,24,26,27]. In accord with this conclusion, the photoreaction of **1a** with **2** using CH_3_COOD as the additive produced mainly mono-deuteriated forms of **3a** and **4a** in which deuterium is present at the stereogenic carbons marked with * in Scheme 4. Therefore, enantioselectivity is governed at the step where protonation of the radical anion takes place.

**Scheme 5:**
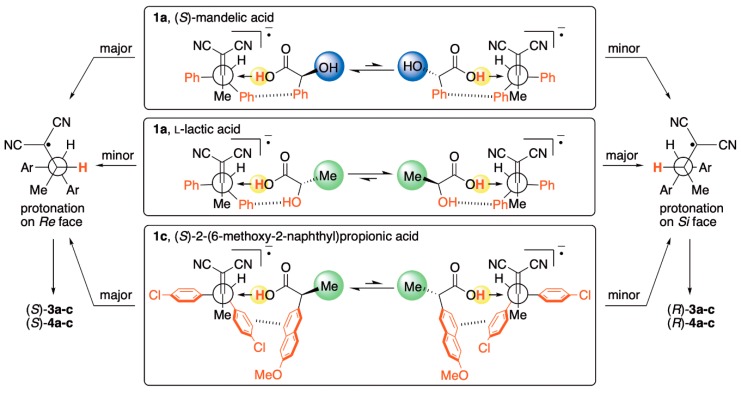
Plausible explanation for the enantioselectivity.

The stereochemistry of protonation of the radical anion **1**^•–^ can be discussed using a Felkin-Anh model (Scheme 5) [31,32,33]. Specifically, in reaction of **1a** in the presence of (*S*)-mandelic acid, proton transfer to the *Re* face of **1a**^•–^ should be preferred in a complex in which a π-π stabilizing interaction occurs between the phenyl groups and the OH group of the acid is located in a sterically less hindered position. Proton transfer to the *Re* face of **1a** leads to the eventual formation of (*S*)-**3a** and (*S*)-**4a**. On the other hand, in the reaction of **1a** in the presence of l-lactic acid, an OH-π interaction between the OH group of the acid and the phenyl group of **1a**^•–^ takes place to form a complex in which proton transfer from the carboxylic acid group occurs preferentially to the *Si* face to minimize steric repulsion of methyl group. This process then gives rise to formation of (*R*)-**3a** and (*R*)-**4a**. In photoreaction of **1c** in the presence of (*S*)-2-(6-methoxy-2-naphthyl)propionic acid, the main enantiomers produced were the same as those generated in reaction of **1c** in the presence of (*S*)-mandelic acid, and %ee increased. This outcome might be a consequence of a strong π-π interaction between the chlorophenyl and the methoxynaphthyl groups.

## 3. Experimental Section

### 3.1. Materials and Equipments

THF was distilled from CaH_2_ and then from Na/benzophenone. CH_3_CN was distilled from P_2_O_5_ then from Ca(OH)_2_. Hexane and 2-propanol were distilled without using a drying agent. Allyltrimethylsilane (**2**) was prepared using a reported procedure [27]. Activated alumina was dried at 200 °C for 2 h before use. Most other chemical substances were used after purification by distillation or recrystallization.

Column chromatography was conducted by using Wakogel C-70~230 (Fujifilm Wako Pure Chemical Corporation, Osaka, Japan). Thin-layer chromatography was performed by using Merck Kiesel gel 60 F_254_ plates (Merck KGaA, Darmstadt, Germany). HPLC separations (achiral) were performed on a recycling preparative HPLC equipped with Jasco PU-987 pump, UV-970 UV detector, and a Chemcosorb I-5Si column (Chemco Plus Scientific Co., Ltd., Osaka, Japan) using hexane-AcOEt or hexane-2-propanol as an eluent, or a recycling preparative HPLC equipped with Jasco PU-2086 pump, RI-2031 differential refractometer (Jasco Corporation, Tokyo, Japan), and Megapak GEL 201F columns (GPC) using CHCl_3_ as an eluent (Jasco Corporation, Tokyo, Japan).

^1^H and ^13^C-NMR spectra were recorded using a Varian MERCURY-300 (300 MHz and 75 MHz, respectively, (Varian Inc., Palo Alto, CA, USA) spectrometer with Me_4_Si as an internal standard. Mass spectra (EI, achiral) were recorded on a SHIMADZU GCMS-QP5050 (Shimadzu Corporation, Kyoto, Japan) operating in the electron impact mode (70 eV) equipped with GC-17A and DB-5MS column (J&W Scientific Inc., Serial: 8696181, Folsom, CA, USA). UV-vis spectra were recorded using a Jasco V-530 spectrophotometer (Jasco Corporation, Tokyo, Japan).

### 3.2. Preparation of **1a**

A mixture of 1,1-diphenylacetone (1.051 g, 5.0 mmol), malononitrile (0.330 g, 5.0 mmol) and activated alumina (1.5 g) was stirred at 60 °C for 1 h [34]. The solids were removed by filtration. Concentration of the filtrate gave a residue that was subjected to silica gel column chromatography followed by recrystallization from hexane to give 2-(1,1-diphenylpropan-2-ylidene)malononitrile (**1a**, white solid, 0.531 g, 2.06 mmol, 41% yield). Lit [35].

### 3.3. Preparation of **1b**

A THF (50 mL) solution of 4-bromoanisole (17.53 mL, 140.0 mmol) was added dropwise to stirred Mg turnings (3.889 g, 160.0 mmol). A small amount of I_2_ was added to facilitate the reaction. A THF (20 mL) solution of ethyl l-lactate (4.587 mL, 40.0 mmol) was added dropwise to the solution, and the resulting mixture was stirred at reflux, cooled, and extracted with Et_2_O and NH_4_Cl aq [36]. The organic layer was dried over Na_2_SO_4_, filtered, and concentrated in vacuo, giving a residue that was subjected to silica gel column chromatography to give 1,1-bis(4-methoxyphenyl)propane-1,2-diol (5.76 g, 20.0 mmol, 50% yield, including inpurity).

25% H_2_SO_4_ aq (15 mL) was added to stirred 1,1-bis(4-methoxyphenyl)propane-1,2-diol (5.76 g, 20.0 mmol, including inpurity), and the resulting solution was stirred at reflux for 3.5 h, cooled, neutralized with Na_2_CO_3_ and extracted with Et_2_O [36]. The organic layer was dried over Na_2_SO_4_, filtered, and concentrated in vacuo, giving a residue that was subjected to silica gel column chromatography to give 1,1-bis(4-methoxyphenyl)propan-2-one (1.047 g, 3.87 mmol, 19% yield).

A mixture of 1,1-bis(4-methoxyphenyl)propan-2-one (1.047 g, 3.87 mmol), malononitrile (0.384 g, 5.82 mmol) and activated alumina (3.0 g) was stirred at 90 °C for 1.5 h [34]. The solids were removed by filtration. Concentration of the filtrate gave a residue that was subjected to HPLC to give 2-[1,1-bis(4-methoxyphenyl)propan-2-ylidene]malononitrile (**1b**, 0.728 g, 2.29 mmol, 59% yield). ^1^H-NMR (300 MHz, CDCl_3_) δ 2.21 (s, 3H), 3.82 (s, 6H), 5.59 (s, 1H), 6.89 (d, *J* = 8.6 Hz, 4H), 7.05 (d, *J* = 8.6 Hz, 4H) ppm.

### 3.4. Preparation of **1c**

A THF (12 mL) solution of 4-bromochlorobenzene (6.647 g, 34.7 mmol) was added dropwise to stirred Mg turnings (0.729 g, 30.0 mmol). A small amount of I_2_ was added to facilitate the reaction. A THF (5 mL) solution of ethyl l-lactate (1.247 mL, 10.9 mmol) was added dropwise to the solution, and the resulting solution was stirred at reflux, cooled, and extracted with Et_2_O and NH_4_Cl aq [36]. The organic layer was dried over Na_2_SO_4_, filtered, and concentrated in vacuo, giving a residue that was subjected to silica gel column chromatography to give 1,1-bis(4-chlorophenyl)propane-1,2-diol (4.023 g, including inpurity).

To stirred 1,1-bis(4-chlorophenyl)propane-1,2-diol (4.023 g, including inpurity) was added 25% H_2_SO_4_ aq (12 mL), and the resulting solution was stirred at reflux for 3.5 h, cooled, neutralized with Na_2_CO_3_ and extracted with Et_2_O [36]. The organic layer was dried over Na_2_SO_4_, filtered, and concentrated in vacuo, giving a residue that was subjected to silica gel column chromatography to give 1,1-bis(4-chlorophenyl)propan-2-one (0.934 g, 3.36 mmol, 31% yield (two steps)). ^1^H-NMR (300 MHz, CDCl_3_) δ 2.24 (s, 3H), 5.05 (s, 1H), 7.11-7.32 (m, 8 H) ppm.

A mixture of 1,1-bis(4-chlorophenyl)propan-2-one (0.934 g, 3.36 mmol), malononitrile (0.444 g, 6.72 mmol) and activated alumina (3.0 g) was stirred at 90 °C for 1.5 h [34]. The solids were removed by filtration. Concentration of the filtrate gave a residue that was subjected to HPLC to give 2-[1,1-bis(4-chlorophenyl)propan-2-ylidene]malononitrile (**1c**, 0.602 g, 1.84 mmol, 55% yield). ^1^H-NMR (300 MHz, CDCl_3_) δ 2.21 (s, 3H), 5.62 (s, 1H), 7.05 (d, *J* = 8.4 Hz, 4H), 7.37 (d, *J* = 8.4 Hz, 4H) ppm; ^13^C-NMR (75 MHz, CDCl_3_) δ 21.33, 55.87, 88.80, 111.37, 111.49, 129.47, 129.94, 134.47, 135.75, 179.56 ppm; MS (EI) *m*/*z* (relative intensity, %) = 114 (52), 139 (89), 165 (59), 291 (100), 326 (62, M^+^).

### 3.5. General Procedure for Photoreactions

CH_3_CN (8 mL) solutions of a 3,3-diaryl-1,1-dicyano-2-methylprop-2-ene (**1a**–**c**, 0.14 mmol), allyltrimethylsilane (**2**, 0.42 mmol), phenanthrene (0.07 mmol), and CH_3_COOH (1 mL) or a chiral carboxylic acid (0.14 mmol) in Pyrex vessels were degassed by argon bubbling for 5 min and then the vessels were sealed. The solutions were irradiated by using a 300 W high pressure mercury lamp (Eikosha, EHB-W-300 or PIH-300) for 2–24 h at room temperature, maintained by using circulated cooling water. The photolysates were extracted with Et_2_O. The organic layer was washed with H_2_O, dried over Na_2_SO_4_, filtered, and concentrated in vacuo, giving a residue that was subjected to HPLC to give **3a**–**c** and **4a**–**c**.

2-Allyl-2-(1,1-diphenylpropan-2-yl)malononitrile (**3a**): ^1^H-NMR (300 MHz, CDCl_3_) δ 1.20 (d, *J* = 6.8 Hz, 3H), 2.42–2.59 (m, 2H), 2.91–3.02 (m, 1H), 4.16 (d, *J* = 9.3 Hz, 1H), 5.28 (d, *J* = 17.6 Hz, 1H), 5.37 (d, *J* = 9.6 Hz, 1H), 5.76-5.92 (m, 1H), 7.21–7.40 (m, 10H) ppm; MS (EI) *m*/*z* = 41, 65, 77, 91, 102, 115, 128, 151, 165, 167, 193, 300 (M^+^).

2-Allyl-2-[1,1-bis(4-methoxyphenyl)propan-2-yl]malononitrile (**3b**): ^1^H-NMR (300 MHz, CDCl_3_) δ 1.18 (d, *J* = 6.7 Hz, 3H), 2.44-2.60 (m, 2H), 2.80–2.91 (m, 1H), 3.76 (s, 3H), 3.78 (s, 3H), 4.08 (d, *J* = 9.2 Hz, 1H), 5.29 (d, *J* = 16.9 Hz, 1H), 5.36 (d, *J* = 10.0 Hz, 1H), 5.77–5.91 (m, 1H), 6.81–6.87 (m, 4H), 7.19–7.26 (m, 4H) ppm.

2-Allyl-2-[1,1-bis(4-chlorophenyl)propan-2-yl]malononitrile (**3c**): ^1^H-NMR (300 MHz, CDCl_3_) δ 1.18 (d, *J* = 6.7 Hz, 3H), 2.52 (dd, *J* = 13.8, 7.5 Hz, 1H), 2.62 (dd, *J* = 13.9, 6.7 Hz, 1H), 2.88 (dq, *J* = 8.9, 6.7 Hz, 1H), 4.15 (d, *J* = 9.1 Hz, 1H), 5.31 (d, *J* = 16.9 Hz, 1H), 5.40 (d, *J* = 10.2 Hz, 1H), 5.76–5.91 (m, 1H), 7.19–7.36 (m, 8H) ppm; ^13^C-NMR (75 MHz, CDCl_3_) δ 15.47, 41.40, 42.40, 42.79, 54.31, 114.18, 123.29, 128.44, 129.24, 129.27, 129.67, 129.93, 133.41, 133.76, 139.09, 139.24 ppm.

2-(1,1-Diphenylpropan-2-yl)malononitrile (**4a**): ^1^H-NMR (300 MHz, CDCl_3_) δ 1.30 (d, *J* = 6.6 Hz, 3H), 2.95–3.06 (m, 1H), 3.64 (d, *J* = 3.3 Hz, 1H), 3.80 (d, *J* = 11.7 Hz, 1H), 7.21–7.36 (m, 10H) ppm; MS (EI) *m*/*z* = 51, 63, 77, 83, 102, 128, 151, 165, 167, 193, 300 (M^+^).

2-[1,1-Bis(4-methoxyphenyl)propan-2-yl]malononitrile (**4b**): ^1^H-NMR (300 MHz, CDCl_3_) δ 1.28 (d, *J* = 6.6 Hz, 3H), 2.82–2.95 (m, 1H), 3.66 (d, *J* = 3.3 Hz, 1H), 3.69 (d, *J* = 11.7 Hz, 1H), 3.77 (s, 6H), 6.83–6.89 (m, 4H), 7.18–7.24 (m, 4H) ppm.

2-[1,1-Bis(4-chlorophenyl)propan-2-yl]malononitrile (**4c**): ^1^H-NMR (300 MHz, CDCl_3_) δ 1.29 (d, *J* = 6.6 Hz, 3H), 2.87-2.98 (m, 1H), 3.60 (d, *J* = 3.4 Hz, 1H), 3.78 (d, *J* = 11.8 Hz, 1H), 7.18–7.37 (m, 8H) ppm.

### 3.6. Resolution of Enantiomers

Resolutions of enantiomers of **3a** and **4a** were performed on a recycling preparative HPLC equipped with Jasco PU-980 pump, Jasco UV-970 and CD-2095 detectors (Jasco Corporation, Tokyo, Japan), Daicel CHIRALCEL OJ (**3a**) or OJ-H (**4a**) columns (Daicel Corporation, Osaka, Japan). Eluents were hexane:2-propanol = 7:3 (**3a**) or 9:1 (**4a**). [**3a**] = 0.068 M, [**4a**] = 0.078 M. Both **3a** and **4a** were detected by UV and CD detectors at 270 nm.

Resolutions of enantiomers of **3c** and **4c** were performed by using a SHIMADZU GCMS-QP5050 (Shimadzu Corporation, Kyoto, Japan) operating in the electron impact mode (70 eV) equipped with SUPELCO GAMMA DEXTM 225 column (Sigma-Aldrich Co., LLC, St. Louis, MO, USA). Detector temp = 215 °C, injection temp = 220 °C, inlet pressure = 93.7 kPa, flow rate = 1.0mL/min, linear velocity = 28.1 cm/s, split ratio = 50, carrier gas = N_2_.

## 4. Conclusions

In summary, we found that photoreactions of prochiral 3,3-diaryl-1,1-dicyano-2-methylprop-1-enes **1a**–**c** with allyltrimethylsilane, carried in the presence of enantiomerically pure chiral carboxylic acids, generates photoallylation and photoreduction products with low but finite levels of enantioselectivity. The percent enantiomeric excesses in the products of the process was highest (4.8 %ee) when (*S*)-mandelic acid was used. Enantioselectivities in these reactions are a consequence of sterically governed asymmetric proton transfer in intermediate complexes formed by π-π and OH-π interactions between radical anions of the prochiral alkenes and the chiral carboxylic acids.

## Figures and Tables

**Table 1:** Photoallylation and photoreduction of **1a–c** by using allyltrimethylsilane (**2**) ^a^.

Entry	Substrate	Additive	Irradiation Time/h	Yields/%	
				3	4
1	**1a** (Ar = Ph)	none	4	0 ^b^	53 ^b^
2	**1a** (Ar = Ph)	acetic acid ^c^	4	34 ^b^	31 ^b^
3	**1b** (Ar = *p*-MeOC_6_H_4_)	none	24	0 ^d^	44 ^d^
4	**1b** (Ar = *p*-MeOC_6_H_4_)	acetic acid ^c^	24	20 ^d^	46 ^d^
5	**1c** (*p*-ClC_6_H_4_)	none	2	0 ^d^	36 ^d^
6	**1c** (*p*-ClC_6_H_4_)	acetic acid ^c^	2	33 ^d^	47 ^d^

^a^ Conditions: **1a**–**c** (0.14 mmol), **2** (0.42 mmol), phenanthrene (0.07 mmol), CH_3_CN (8 mL), 300 W high-pressure mercury lamp, Pyrex, r.t. ^b^ Determined by using GC. ^c^ 1 mL. ^d^ Determined by using ^1^H-NMR.

**Table 2:** Enantioselective photoallylations and photoreductions of **1a**–**c** ^a^.

Entry	Substrate	Additive		Irradiation Time/h	Yields/% (ee/%)	
					3	4
1	1a (Ar = Ph)	none		**4**	0 ^b^	53 ^b^
2	**1a** (Ar = Ph)	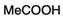	acetic acid^c^	4	34 ^b^	31 ^b^
3	**1a** (Ar = Ph)	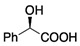	(*R*)-mandelic acid	4	34 ^b^ (+1.5 ^d,e^)	27 ^b^ (+4.1 ^d,e^)
4	**1a** (Ar = Ph)	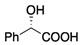	(*S*)-mandelic acid	4	22 ^b^ (−3.4 ^d,f^)	26 ^b^ (−4.8 ^d,f^)
5	**1a** (Ar = Ph)	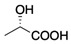	l-lactic acid	4	28^b^ (+0.6 ^d,e^)	39 ^b^ (+3.2 ^d,e^)
6	**1a** (Ar = Ph)	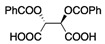	dibenzoyl l-tartaric acid	4	31 ^b^ (−2.0 ^d,f^)	16 ^b^ (−3.6 ^d,f^)
7	**1b** (Ar = *p*-MeOC_6_H_4_)	none		24	0 ^g^	44 ^g^
8	**1b** (Ar = *p*-MeOC_6_H_4_)	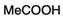	acetic acid ^c^	24	20 ^g^	46 ^g^
9	**1b** (Ar = *p*-MeOC_6_H_4_)	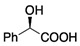	(*R*)-mandelic acid	24	54 ^g^ (nd ^h^)	28^g^ (nd ^h^)
10	**1b** (Ar = *p*-MeOC_6_H_4_)	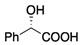	(*S*)-mandelic acid	24	13 ^g^ (nd ^h^)	48^g^ (nd ^h^)
11	**1c** (*p*-ClC_6_H_4_)	none		2	0 ^g^	36 ^g^
12	**1c** (*p*-ClC_6_H_4_)	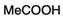	acetic acid ^c^	2	33 ^g^	47 ^g^
13	**1c** (*p*-ClC_6_H_4_)	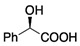	(*R*)-mandelic acid	2	27 ^g^ (+2.0 ^i,j^)	55 ^g^ (+2.0 ^i,j^)
14	**1c** (*p*-ClC_6_H_4_)	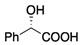	(*S*)-mandelic acid	2	24 ^g^ (−2.6 ^i,k^)	61 ^g^ (−0.6 ^i,k^)
15	**1c** (*p*-ClC_6_H_4_)	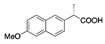	(*S*)-2-(6-methoxy-2-naphthyl)propionic acid	2	0 ^g^	68^g^ (−3.5 ^i,k^)

^a^ Conditions: **1a–c** (0.14 mmol), **2** (0.42 mmol), phenanthrene (0.07 mmol), CH_3_CN (8 mL), additive (0.14 mmol), 300 W high-pressure mercury lamp, Pyrex, r.t. ^b^ Determined by using GC. ^c^ 1 mL. ^d^ Determined by using chiral HPLC. ^e^ Major isomer corresponds to the second peak in the HPLC chart. ^f^ Major isomer corresponds to the first peak in the HPLC chart. ^g^ Determined by using ^1^H-NMR. ^h^ Ee could not be determined. ^i^ Determined by using chiral GC. ^j^ Major isomer corresponds to the second peak in the GC chart. ^k^ Major isomer corresponds to the first peak in the GC chart.

## Supplementary Materials

The following are available online: ^1^H-NMR spectra of **1b**, **1c**, **3a**, **3b**, **3c**, **4a**, and **4c**, ^13^C-NMR spectra of **1c** and **3c**.
