# Supplementary material for: Enantioselective Protonation of Radical Anion Intermediates in Photoallylation and Photoreduction Reactions of 3,3-Diaryl-1,1-dicyano-2-methylprop-1-ene with Allyltrimethylsilane
Source: Molecules. 2019 Jul 23;24(14):2677. doi: 10.3390/molecules24142677 (PMC6680834; doi:10.3390/molecules24142677)
Supplement: Supplementary file 1 [file molecules-24-02677-s001.pdf]

Supporting Information for

Enantioselective Protonation of Radical Anion Intermediates in Photoallylation and Photoreduction Reactions of 3,3-Diaryl-1,1-dicyano-2-methylprop-1-ene with Allyltrimethylsilane

Hajime Maeda *, Masayuki Iida, Daisuke Ogawa and Kazuhiko Mizuno *

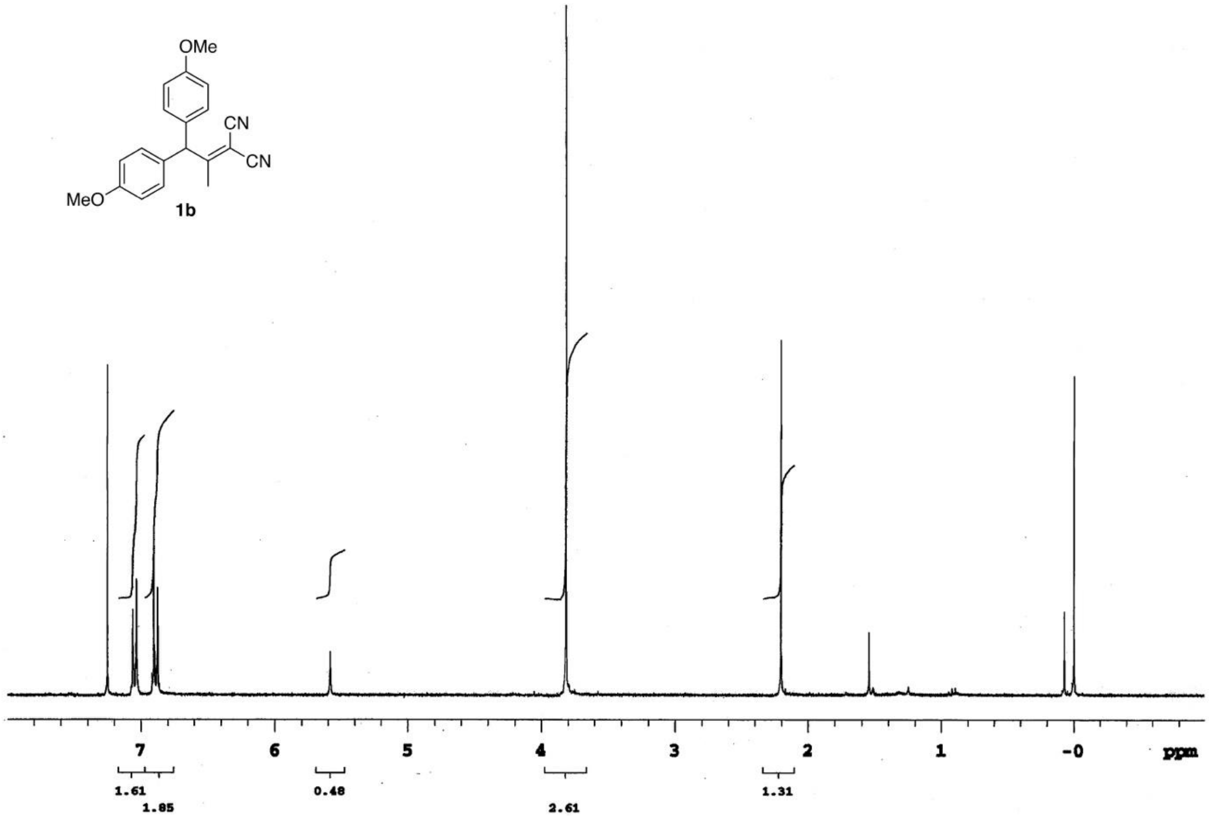

Figure S1. 300 MHz ^1H NMR spectrum of **1b** in $CDCl_3$.

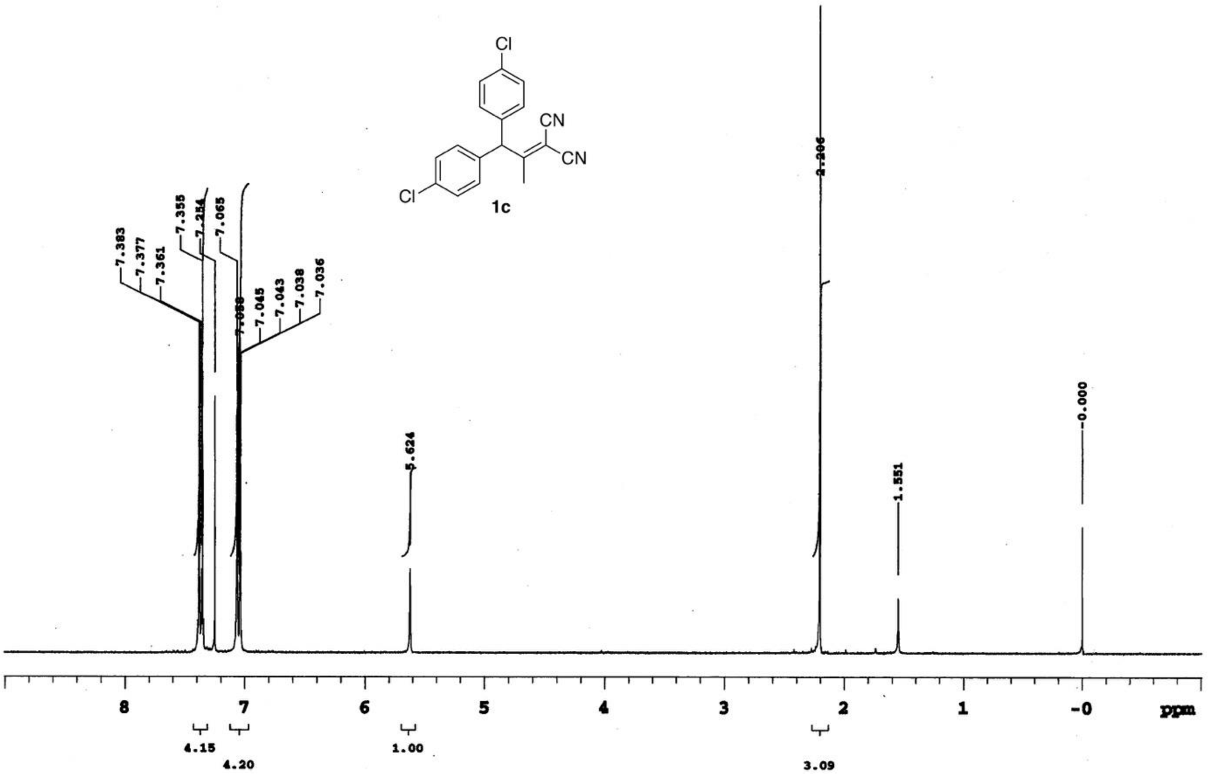

Figure S2. 300 MHz ^{1}H NMR spectrum of **1c** in $CDCl_3$.

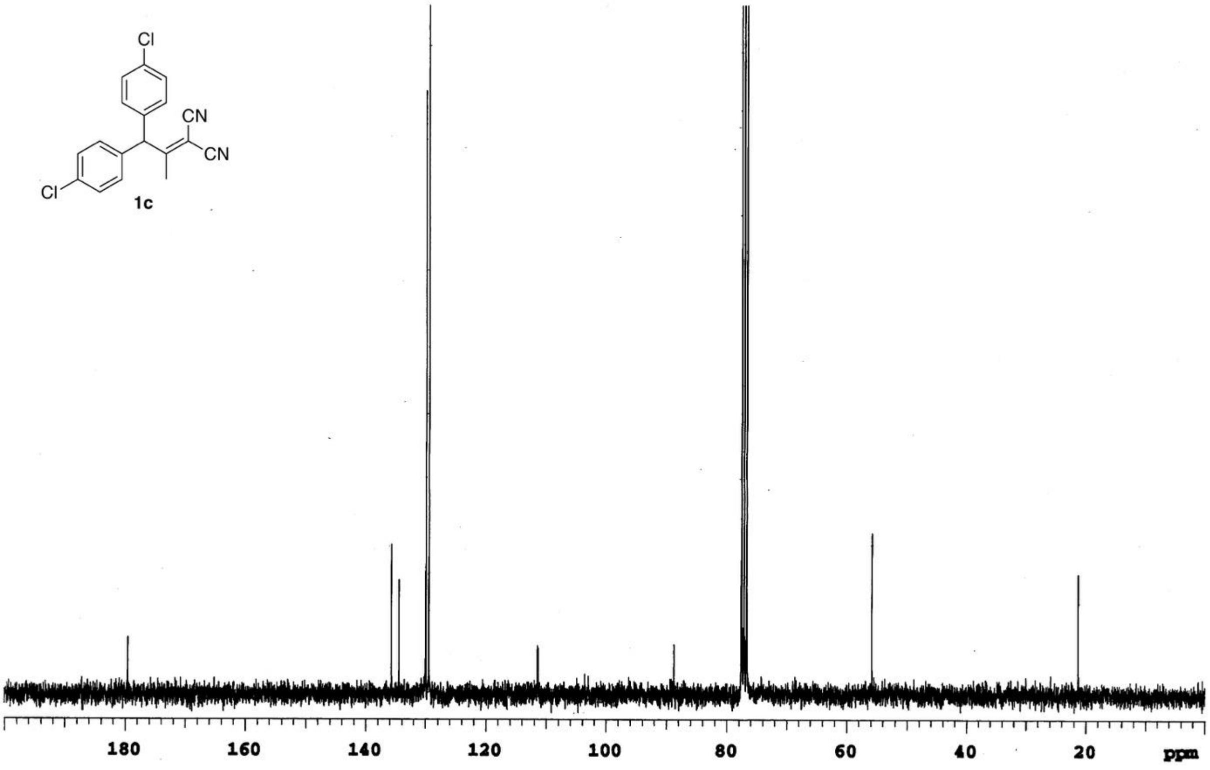

Figure S3. 75 MHz ^{13}C NMR spectrum of **1c** in $CDCl_3$.

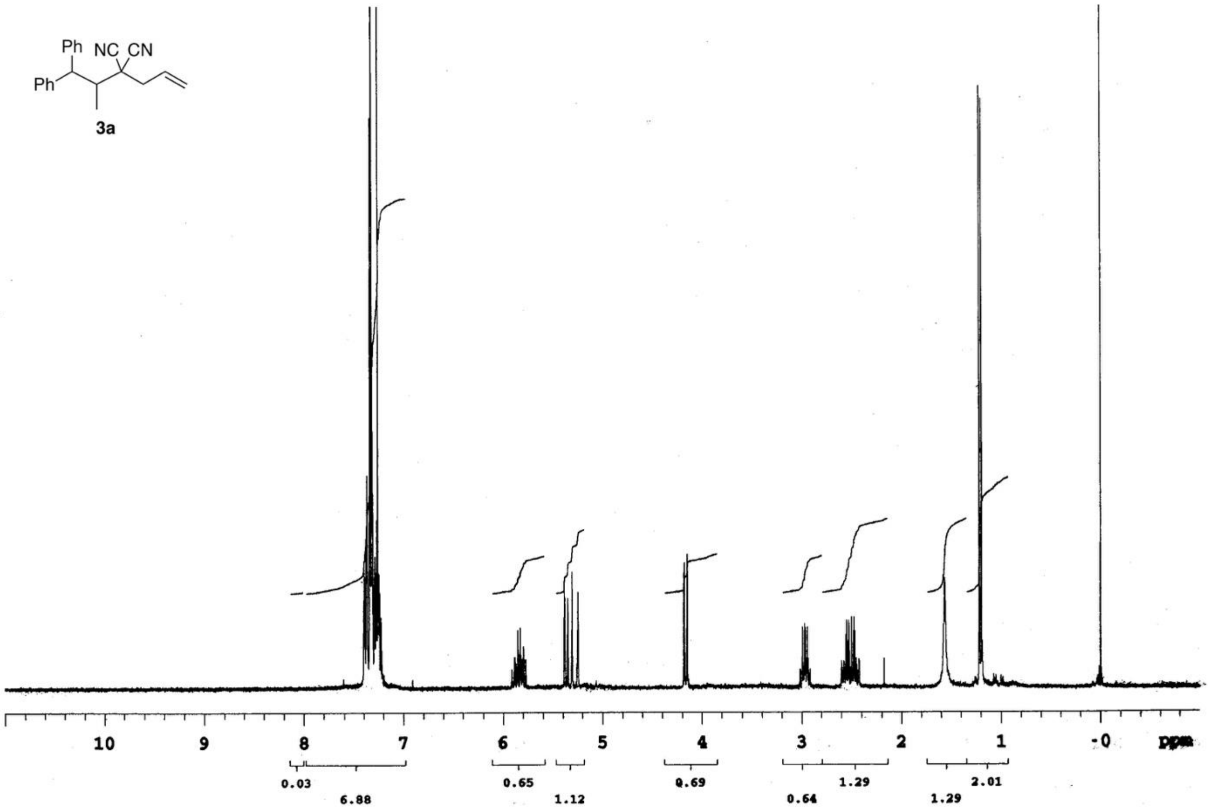

Figure S4. 300 MHz ^{1}H NMR spectrum of **3a** in $CDCl_3$.

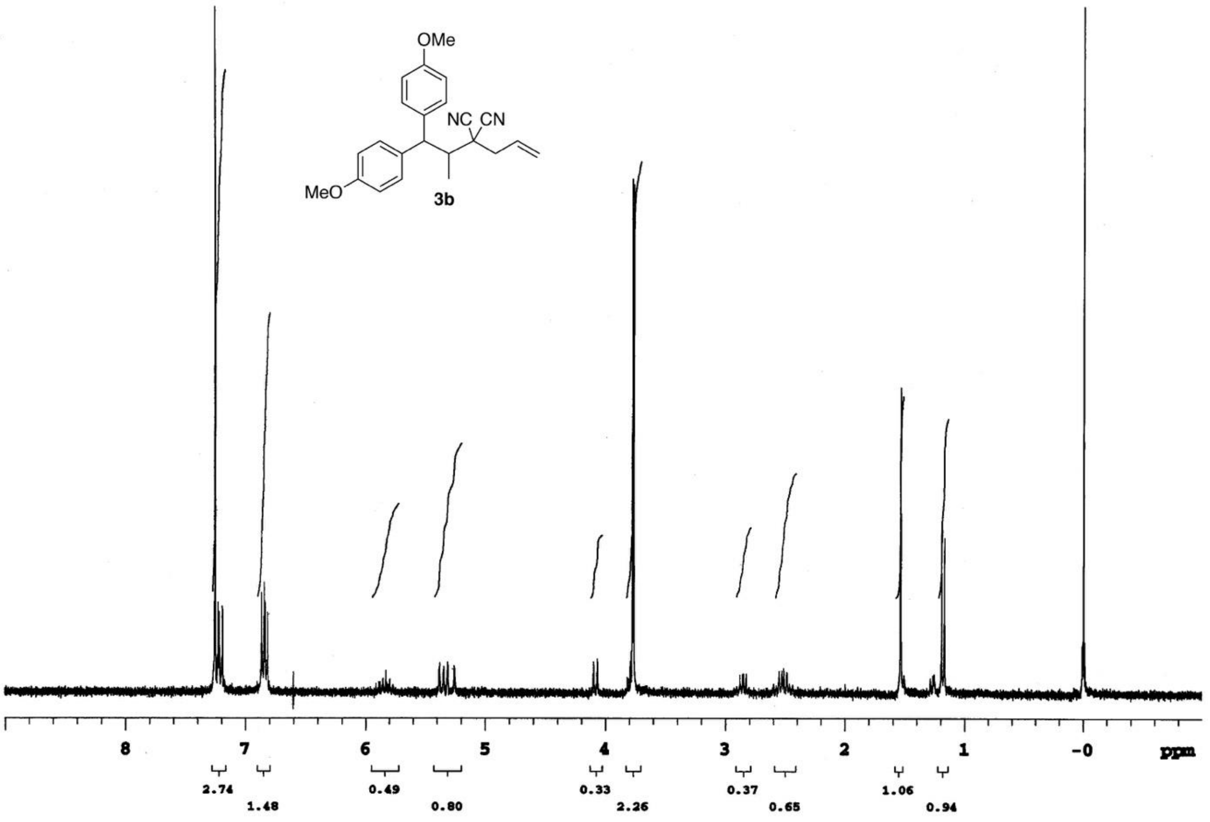

Figure S5. 300 MHz ^{1}H NMR spectrum of **3b** in $CDCl_3$.

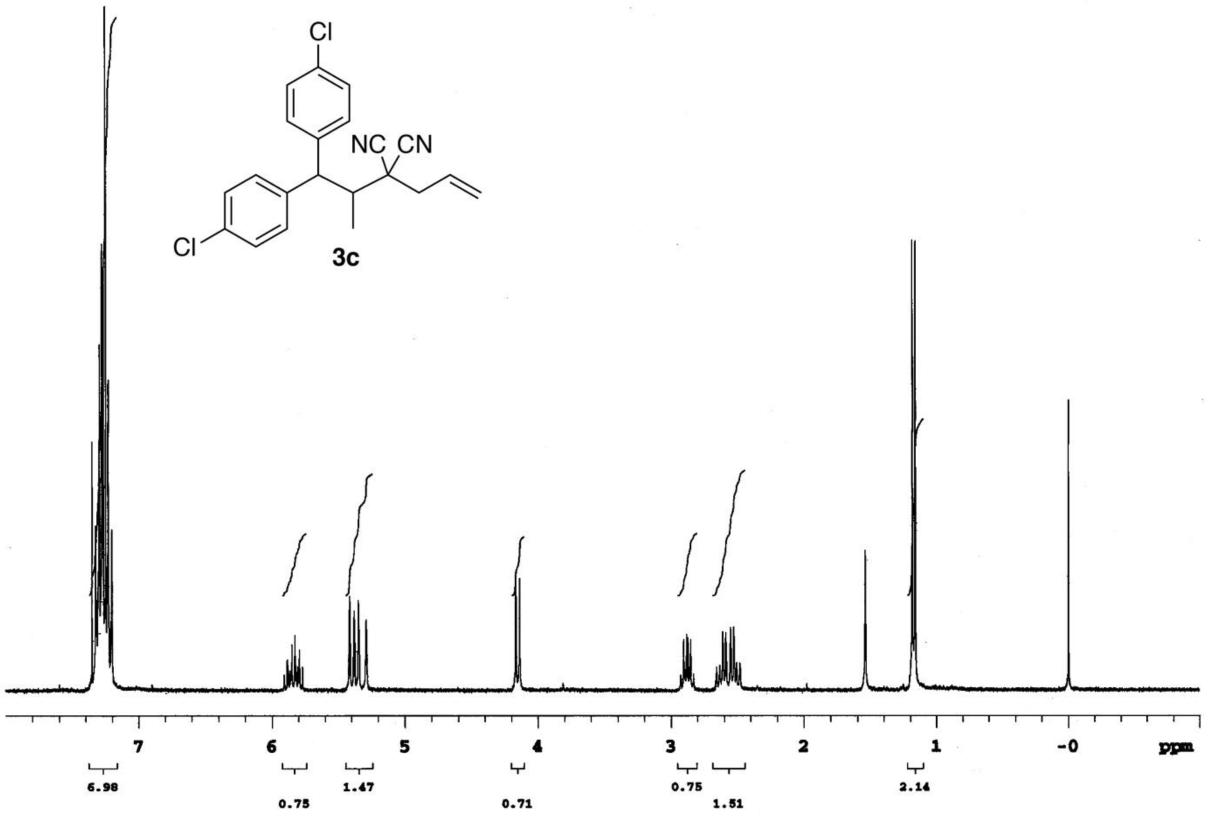

Figure S6. 300 MHz ^{1}H NMR spectrum of **3c** in $CDCl_3$.

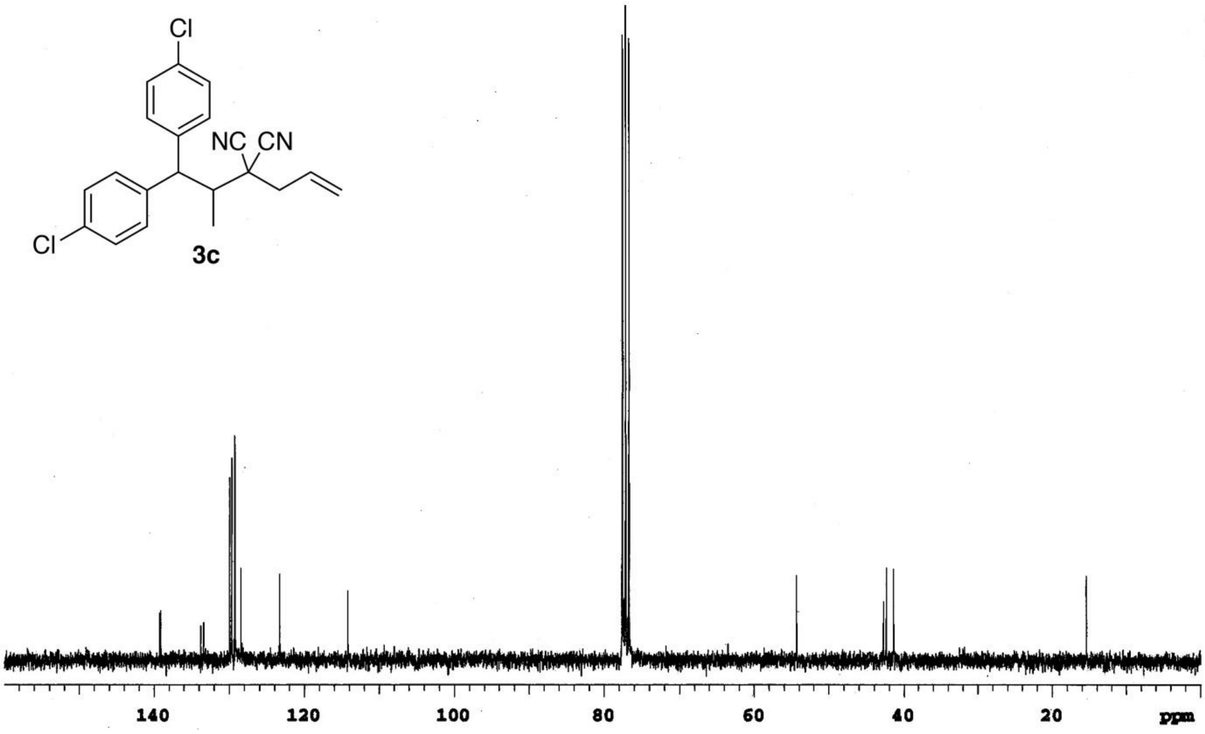

Figure S7. 75 MHz ^{13}C NMR spectrum of **3c** in $CDCl_3$.

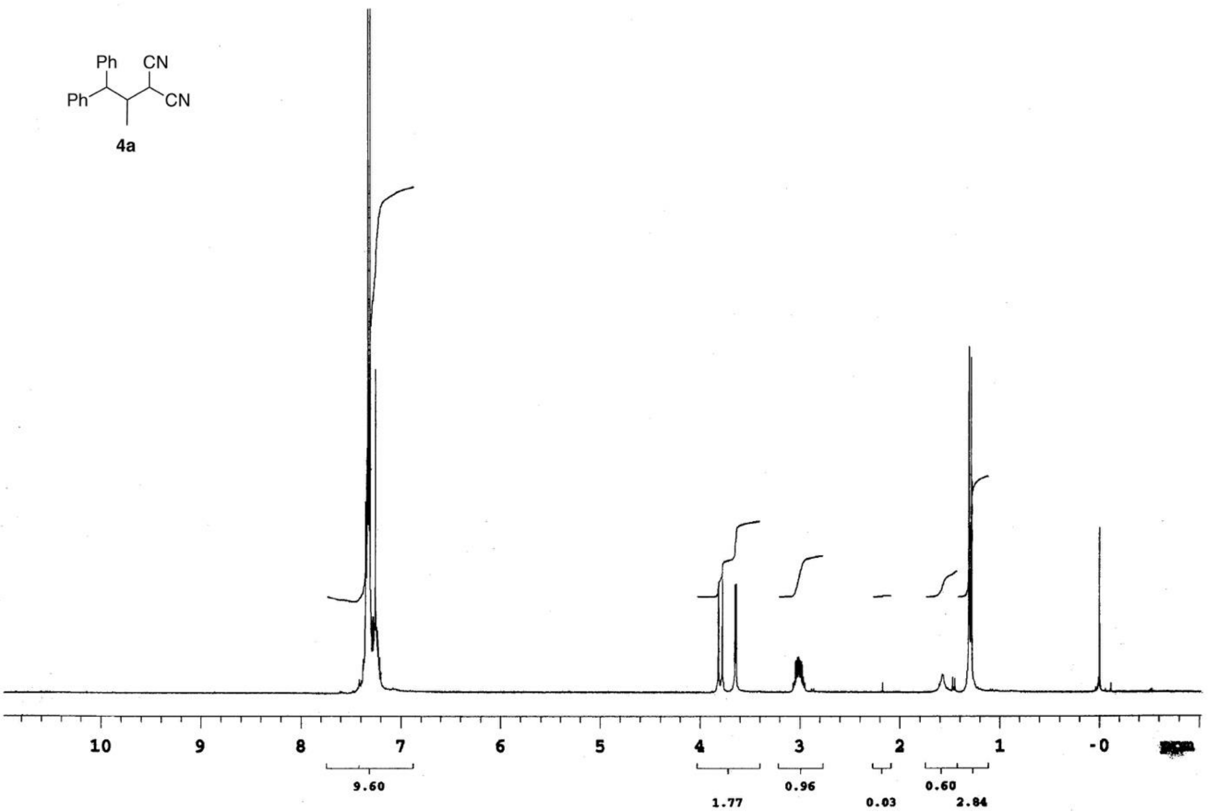

Figure S8. 300 MHz ^{1}H NMR spectrum of **4a** in $CDCl_3$.

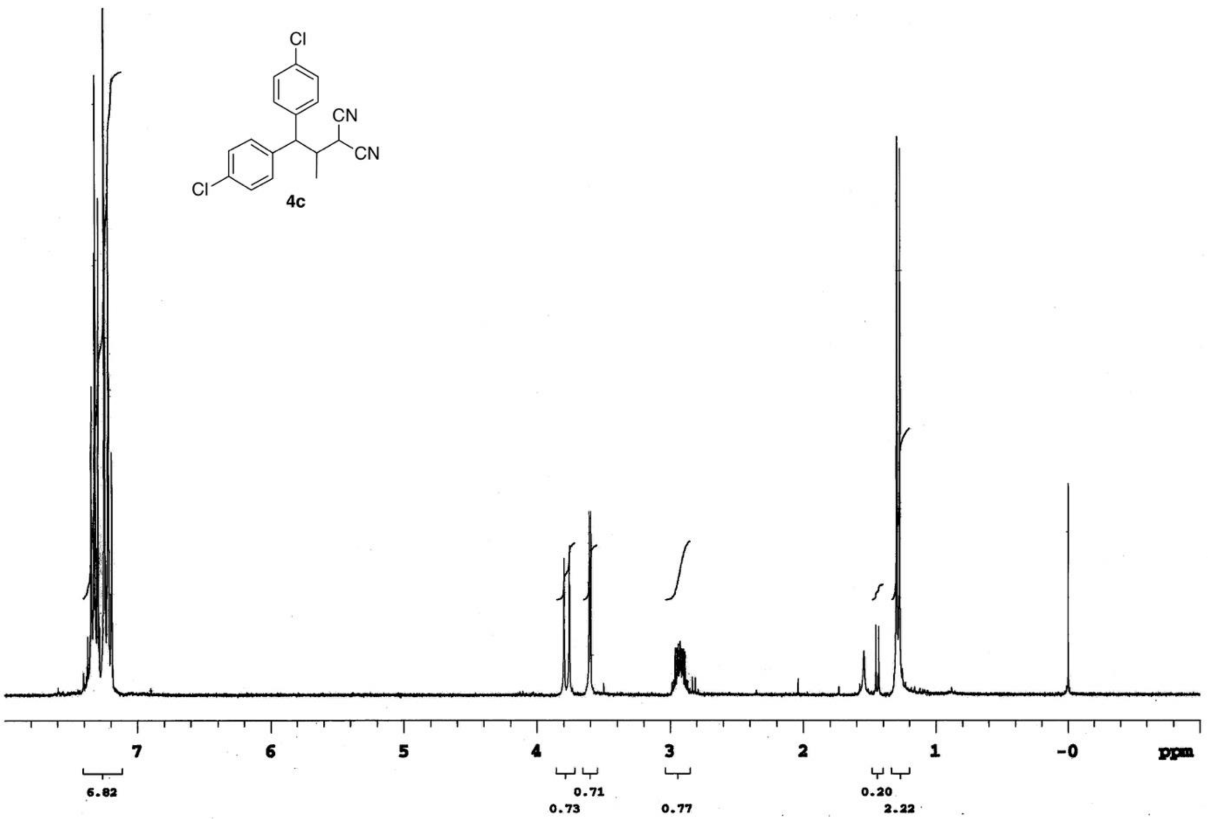

Figure S9. 300 MHz ^{1}H NMR spectrum of **4c** in $CDCl_3$.
